# Evaluation of the concordance in HPV type between self- and physician-collected samples using a brush-based device and a PCR-based HPV DNA test in Japanese referred patients with abnormal cytology or HPV infection

**DOI:** 10.1007/s10147-020-01727-5

**Published:** 2020-06-24

**Authors:** Toshimichi Onuma, Tetsuji Kurokawa, Akiko Shinagawa, Yoko Chino, Yoshio Yoshida

**Affiliations:** Department of Obstetrics and Gynecology, Faculty of Medical Sciences, University of Fukui, 23-3 Matsuoka-Shimoaizuki, Eiheiji-cho, Fukui, 910-1193 Japan

**Keywords:** Cervical cancer, Screening, HPV self-sampling, Brush, PCR-based HPV test

## Abstract

**Background:**

To adopt HPV self-sampling in Japan, we assessed the concordance between self- and physician-collected human papillomavirus (HPV) samples from Japanese patients and examined the performance of HPV self-sampling for cervical intraepithelial neoplasia grade 2 or worse (CIN2+).

**Methods:**

Patients who had previously tested negative for intraepithelial lesions or malignancy/HPV-positive, and patients with atypical squamous cells of undetermined significance or worse (ASCUS+) cytology were eligible for this cross-sectional study. Participants performed HPV self-sampling using an Evalyn brush, which was submitted at the Fukui Prefectural Health Care Association. The Evalyn brush heads were stored in ThinPrep vials. The physician, however, performed HPV and cell sampling using an endocervical brush and immediately stored the brush heads in ThinPrep vials. All participants underwent colposcopy and biopsy. Histopathological diagnoses were made by pathologists at Fukui University Hospital. HPV infection was confirmed using a PCR-based Cobas 4800 HPV DNA test. Cytological analysis was performed at Fukui Prefectural Health Care Association.

**Results:**

HPV-positive rates for physician-collected samples and self-collected samples were 51 and 50%, respectively. The perfect match rate of HPV type between the groups was 88% (*κ* = 0.76). HPV16/18 showed higher agreement rates than other HPVs (99%, kappa 0.96 and 89% kappa 0.77, respectively). Both groups showed 100% sensitivity to CIN2+, but specificity was 57.0 and 58.1%, respectively.

**Conclusion:**

For HPV typing, a good concordance rate was seen between self- and physician-collected samples. Self-sampling showed high sensitivity for CIN2+. Self-sampling using the Evalyn brush and Cobas 4800 may be feasible for screening Japanese individuals.

## Introduction

Japan has a higher incidence of age-adjusted cervical cancer than the United States and Australia, where screenings are organized [1]. The mortality rate of cervical cancer patients aged  <50 years is increasing, with the high incidence attributable to the low human papillomavirus (HPV) vaccination rate and low screening rate in Japan [2]. Most cervical cancers occur in unscreened women [3]; therefore, it is necessary to increase the number of women screened for cervical cancer to reduce its incidence in Japan.

Epidemiological studies using molecular technology show that persistent infection with high-risk HPV (hrHPV) is strongly associated with development of cervical intraepithelial neoplasia (CIN) and cervical cancer [4]. Therefore, HPV testing has been introduced alone or combined with cytology for cervical cancer screening. Physician-collected hrHPV tests are more sensitive for detecting CIN2 or worse (CIN2+) than cytology alone [5]. Women who were physician-collected HPV− at baseline have lower rates of CIN2+ at 48 months relative to cytology negative women at baseline [6]. Primary physician-collected HPV screening every 5 years with partial genotyping is predicted to be substantially more effective and potentially cost-saving relative to current cytology screening programs undertaken every 2 years [7]. The Netherlands and Turkey fully implemented national HPV-based cervical cancer screening [8].

HPV self-sampling is a screening method, where HPV sampling is performed by the screening participant and displays high HPV test concordance with HPV physician sampling in various referral and screening populations [9]. HPV self-sampling using polymerase chain reaction (PCR)-based HPV testing is almost equivalent to HPV physician sampling in sensitivity to CIN2+ and displays greater sensitivity for CIN2+ relative to physician-collected cytology [10]. Additionally, HPV self-sampling is less awkward than physician screening and acceptable for screening patients [11]. A previous study of HPV self-sampling for unscreened women showed that self-sampling significantly improved participation in the screening program [12, 13]. Therefore, HPV self-sampling was adopted as part of the cervical cancer screening program in the Netherlands [14].

Several HPV self-sampling devices and detection methods are available [15]. HPV self-sampling with brush- and lavage-based self-collection devices show increased sensitivity for CIN2+ relative to sampling performed with swab- or tampon-based self-collection [10]. Among self-sampling brush devices, the Evalyn brush shows high performance and good patient acceptance [16, 17]. A recent meta-analysis reported that the hybrid capture (HC) method is less sensitive for CIN2+ than the HPV DNA PCR method for HPV self-sampling [15]. To apply self-sampling in clinical practice, it is important to use clinically evaluated PCR-based HPV testing. The Cobas 4800 is a clinically established PCR-based HPV test [18] that shows good HPV test performance for CIN detection in referral populations [19, 20]. However, the HPV concordance rate and CIN2+-detection sensitivity using the Evalyn brush and Cobas 4800 in the Japanese population have not been reported. Furthermore, the study population using these methods involved patients with premalignant lesions, cervical carcinoma, and carcinoma suspicion [19] or patients with atypical squamous cells of undetermined significance (ASCUS) [20]. Additionally, CIN has been detected in negative for intraepithelial lesions or malignancy (NILM)/HPV-positive Japanese patients [21]. Introduction of HPV self-sampling for screening requires confirmation of consistency with results of physician sampling of NILM/HPV-positive patients. However, there are no reports using the Evalyn brush and/or the Cobas 4800 showing concordance rates between self-collection and physician collection in NILM/HPV-positive groups.

We hypothesized that HPV self-sampling with the Evalyn brush and Cobas 4800 PCR-based HPV testing would show good HPV-type agreement between physician sampling and self-sampling along with high detection sensitivity of CIN2+ in the Japanese population. To test this hypothesis, we compared HPV self-sampling using the Evalyn brush and Cobas 4800 with physician sampling performed in a Japanese referral population that included NILM/HPV patients.

## Patients and methods

### Study population

We conducted a cross-sectional study comparing the results of HPV self-sampling with physician sampling at the University of Fukui Hospital from January 2019 to July 2019. Previous studies report that referral populations sampled by physicians showed an hrHPV-infection rate of ~20 to 50%, resulting in colposcopy, regardless of cytology results [9, 15]. To obtain a referral population with the same HPV-infection rate as previous studies, as well as NILM/HPV patients, we included two patient types: (1) outpatients with abnormal cytology and requiring colposcopy and biopsy and (2) NILM/HPV-positive patients in the Fukui Cervical Cancer Study (FCCS). The FCCS investigated whether combined screening with liquid-based cytology (LBC) and HPV testing could be useful in Japan within the framework of actual screening. After an initial visit, NILM/HPV-positive patients in the baseline phase of the FCCS study were followed up for 3 years and consistently underwent physician-collected HPV testing, cytology, and colposcopy at the annual visit, unless they showed CIN3 or worse (CIN3+) [21]. Exclusion criteria included patients who had undergone hysterectomy, were pregnant, or who had received chemotherapy. Patients not excluded provided written informed consent for participation following explanation of the study design. This study was approved by the Fukui University Hospital Ethics Committee (no. 20180080).

### Sample collection

Participants received instruction on how to submit samples after HPV self-sampling but not details concerning use of the Evalyn brush for sample collection. Participants were instructed to read the instructions describing use of the Evalyn brush before self-sampling, with these instructions created under supervision of the Japan Cancer Society. These instructions were verified that Japanese people could read and understand before this study. Participants performed HPV self-sampling in the bathroom at the hospital and then submitted the brushes. Immediately after HPV self-sampling, HPV physician sampling and cytology were performed. The Rovers Cervex brush (Rovers Medical Devices, Oss, The Netherlands) was used for physician sampling. After sampling, the Cervex brush was immediately placed in ThinPrep vials (Hologic, Marlborough, MA, USA), and colposcopy and biopsy were performed. All physicians involved in sampling were gynecologic oncology specialists. The Evalyn brush and physician-sampled ThinPrep vials were stored at room temperature and transferred to the Fukui Health Care Association on a fixed day of the week. Cytologists examined the cytology samples using the LBC method at the Fukui Health Care Association. Two pathologists at Fukui University Hospital examined the colposcopic biopsy tissue. Cytologists and pathologists were not informed of HPV test results prior to diagnosis.

### HPV testing

The Evalyn brush was processed after transfer to the Fukui Health Care Association. The Evalyn brush was placed into ThinPrep vials and stirred to release the cells. Both physician- and self-sampled ThinPrep vials were stored at room temperature until measurement. HPV testing was performed using the Cobas 4800 system. HPV-16 and -18 as well as 12 other HPV genotypes, including -31, -33, -35, -39, -45, -51, -52, -56, -58, -59, -66, and -68, were measured. Previously reported methods were used for all measurements [18, 21]. HPV testing was performed without information on patient background, cytology results, or histology results.

### Statistical analysis

Continuous variables are presented as the mean ± standard deviation, and categorical variables are expressed as frequencies and proportions. Student’s *t* test was used to compare continuous variables. Agreement rates of perfect matches for HPV typing between the self- and physician-collected samples were examined, as were agreement rates for HPV-16/-18 and HPV others. HPV concordance between paired samples was assessed using the Kappa statistic (Cohen’s Kappa; *κ*) and defined as “Poor” (*κ* ≤ 0.20), “Fair” (0.21 ≤ *κ* ≤ 0.40), “Moderate” (0.41 ≤ *κ* ≤ 0.60), “Good” (0.61 ≤ *κ* ≤ 0.80), or “Very good” (*κ* ≥ 0.81). The sensitivity and specificity of HPV self-sampling were calculated using HPV physician sampling as the standard [20]. CIN2+ was defined as CIN2, CIN3, adenocarcinoma (ADC) in situ (AIS), squamous cell carcinoma (SCC), or ADC. CIN2+-detection sensitivity, specificity, positive predictive value (PPV), and negative predictive value (NPV) were calculated for both sample sets. ASCUS+ was defined as ASCUS, low-grade squamous intraepithelial lesion (LSIL), high-grade squamous intraepithelial lesion (HSIL), atypical squamous cells, cannot exclude HSIL (ASCH), SCC, atypical glandular cells (AGCs), and ADC. LSIL+ was defined as LSIL, ASCH, HSIL, SCC, AGC, or ADC. The detection sensitivity, specificity, PPV, and NPV for CIN2+ were calculated for ASCUS+ and LSIL+ patients. Data were analyzed using SPSS (v.21.0; IBM Corp., Armonk, NY, USA), and a *p* < 0.05 was considered significant.

## Results

### Patient characteristics

Table 1 shows the age, cytological results, and histological results of participants. Patients aged 30–39 years were the majority (39%: 39/100), followed by women aged 40–49 years (28%; 28/100), 20–29 years (13%; 13/100), and 50–59 years (13%; 13/100). Two cases produced unsatisfactory Pap results. Cytological results were NILM 72.4% (71/98), ASCUS+ 27.6% (27/98), and LSIL+ 21.4% (21/98). One case did not undergo biopsy without colposcopic lesions. There were 73.7% (73/99) cases without dysplasia, 13.1% (13/99) of CIN1, 13.1% (13/99) of CIN2+, and 7.1% (7/99) of CIN3+.

**Table 1:** Patient characteristics

*n*	100
Average age (SD)	41.8 (11.0)
Cytology	
NILM	71
ASCUS	6
LSIL	6
ASCH	2
HSIL	9
SCC	2
AGC	1
ADC	1
Unsatisfactory Pap test	2
Pathology	
No dysplasia	73
CIN1	13
CIN2	6
CIN3	3
SCC	1
AIS	2
ADC	1
No biopsy	1

*ADC* adenocarcinoma, *AGC* atypical glandular cells, *AIS* adenoma in situ, *ASC-H* atypical squamous cells, cannot exclude HSIL, *ASCUS* atypical squamous cells of undetermined significance, *CIN* cervical intraepithelial neoplasia, *HSIL* high-grade squamous intraepithelial lesion, *LSIL* low-grade squamous intraepithelial lesion, *NILM* negative for intraepithelial lesion or malignancy, *SCC* squamous cell carcinoma, *SD* standard deviation

### HPV type according to self- and physician sampling

Table 2 shows the results of HPV type according to HPV self- and physician sampling. All self- and physician samples were valid for HPV testing. The HPV+ rates for self- and physician sampling were 50.0 and 51.0%, respectively, with similar rates of different HPV types between sampling methods. Cases with multiple HPV infections were 4.0% (2/50) for self-sampling and 5.9% (3/51) for physician sampling, with no cases with HPV-16 or -18 infections found. There were 11 HPV+ or HPV− discrepancies and one case of HPV-type discrepancy.

**Table 2:** HPV type in self- and physician sampling

	pHPV					
	HPV (−)	HPV16	HPV18	HPV others	HPV-16, others	HPV-18, others
sHPV						
HPV (−)	44	0	0	6	0	0
HPV-16	0	4	0	0	0	0
HPV-18	0	0	7	0	0	0
HPV others	5	0	0	31	1	0
HPV-16, others	0	0	0	0	1	0
HPV-18, others	0	0	0	0	0	1

There were no cases of HPV-16 or -18 infection. HPV others includes -31, -33, -35, -39, -45, -51, -52, -56, -58, -59, and -66

*HPV* human papillomavirus, *pHPV* HPV physician sampling, *sHPV* HPV self-sampling

### Performance of HPV self-sampling relative to physician sampling

Table 3 shows the performance of HPV self-sampling relative to physician sampling used as a reference standard. Coincidence was defined as a case when HPV type was the same. The HPV all type showed an agreement rate of 88% (*κ*: 0.76), sensitivity of 86% [95% confidence interval (CI): 74–94%], and specificity of 90% (95% CI 78‒97%). HPV-16/-18 showed a higher agreement rate than HPV others (99.0%, *κ*: 0.96; and 89.0%, *κ*: 0.77, respectively). Additionally, HPV-16/-18 showed higher sensitivity and specificity than HPV others (92.9 and 100%; and 85.4 and 91.5%, respectively).

**Table 3:** Performance of HPV self-sampling relative to physician sampling

	Agreement rate (%)	Kappa (95% CI)	Sensitivity (95% CI)	Specificity (95% CI)
HPV, all types	88.0	0.76 (0.63–0.89)	86.3 (73.7–94.3)	89.8 (77.8–96.6)
HPV-6/18	99.0	0.96 (0.87–1.0)	92.9 (66.1–99.8)	100 (93.8–100)
HPV others	89.0	0.77 (0.65–0.90)	85.4 (70.8–94.4)	91.5 (81.3–97.2)

HPV others includes -31, -33, -35, -39, -45, -51, -52, -56, -58, -59, and -66

CI, confidence interval; HPV, human papillomavirus; pHPV, HPV physician sampling; sHPV, HPV self-sampling

### Discordant cases between self- and physician sampling

Table 4 shows cases with inconsistent results for HPV testing between sampling methods. Overall, 91.7% (11/12) of discordant cases were HPV others or HPV−, and 91.7% (11/12) were NILM. There were no cases with dysplasia among discordant cases, and the average age between concordant and discordant cases did not differ significantly (42.1 ± 11.1 and 39.6 ± 11.1 years, respectively; *p* = 0.461). The number of days from self-sampling to HPV testing in all cases was 12.7 ± 6.9 days for HPV test-matched cases and 14.2 ± 7.3 days for HPV test-unmatched cases, although the difference was not significant (*p* = 0.482).

**Table 4:** Discordant cases between self- and physician sampling

Age (years)	sHPV	pHPV	Cytology	Pathology
29	(−)	Others	NILM	No dysplasia
29	(−)	Others	NILM	No dysplasia
31	(−)	Others	NILM	No dysplasia
35	Others	(−)	NILM	No dysplasia
35	Others	(−)	NILM	No dysplasia
36	(−)	Others	NILM	No dysplasia
36	Others	Others, HPV-16	HSIL	No dysplasia
38	Others	(−)	NILM	No dysplasia
42	(−)	Others	NILM	No dysplasia
44	Others	(−)	NILM	No dysplasia
52	(−)	Others	NILM	No dysplasia
68	Others	(−)	NILM	No dysplasia

Others includes HPV-31, -33, -35, -39, -45, -51, -52, -56, -58, -59, and -66

*HSIL* high-grade squamous intraepithelial lesion, *HPV* human papillomavirus, *NILM* negative for intraepithelial lesion or malignancy, *pHPV* HPV physician sampling, *sHPV* HPV self-sampling

### CIN2+ detection sensitivities of self-sampling, physician sampling, and cytology

Table 5 shows the CIN2+-detection sensitivity of HPV self-sampling, physician sampling, ASCUS+, and LSIL+. Self-sampling showed the same CIN2+-detection sensitivity and specificity as physician sampling (100 and 58.1%; and 100 and 57.0%, respectively). Self-sampling showed the same CIN2+-detection sensitivity but less specificity relative to cytology (self-sampling: 100 and 58.1%; ASCUS+: 100 and 84.5%; and LSIL+: 92.3 and 89.3%). The coincidence rates between self-sampling and physician sampling for CIN2+ cases was 100%, and HPV-16/-18 showed a higher CIN2+ rate than HPV others for self- and physician sampling. HPV-16/-18 with 57.1% (8/14) for CIN2+ physician sampling and 61.5% (8/13) for CIN2+ self-sampling as compared with HPV others at 15.8% (6/38) and 15.4% (6/39) for CIN2+, respectively.

**Table 5:** CIN2+ detection sensitivity of HPV self-sampling, physician sampling, and cytology

	Sensitivity (%)	Specificity (%)	PPV (%)	NPV (%)
sHPV	100 (66.1‒100)	58.1 (47.0‒68.7)	26.5 (14.9‒41.1)	100 (89.6‒100)
pHPV	100 (66.1‒100)	57.0 (45.8‒67.6)	26.0 (14.6‒40.3)	100 (89.4‒100)
ASCUS+	100 (66.1‒100)	84.5 (75.0‒91.5)	50.0 (29.9‒70.1)	100 (92.5‒100)
LSIL+	92.3 (64.0‒99.8)	89.3 (80.6‒95.0)	57.1 (34.0‒78.2)	98.7 (92.9‒100)

*ASCUS+* atypical squamous cells of undetermined significance or worse, *HPV* human papillomavirus, *LSIL+* low-grade squamous intraepithelial lesion or worse, *NPV* negative predictive value, *pHPV* HPV physician sampling, *PPV* positive predictive value, *sHPV* HPV self-sampling

## Discussion

We investigated whether HPV self-sampling with an Evalyn brush and Cobas 4800 demonstrated HPV-type agreement with physician sampling and high detection sensitivity for CIN2+ in a Japanese referral population. The HPV-type concordance rate between sampling methods was 88% (*κ*: 0.76), with HPV-16/-18 showing a higher match rate and *κ* than HPV others. The detection sensitivity for CIN2+ by self-sampling was 100%. The results showed that HPV self-sampling using an Evalyn brush and a Cobas 4800 PCR-based method might be feasible for cervical cancer screening in Japan.

In HPV self-sampling, participants collect the samples, making sample validity dependent on the patient. Therefore, it is important to compare HPV test results with those obtained from physician-collected samples considered the gold standard. The results of the present study showed a complete agreement rate for HPV typing of 88% (*κ*: 0.76), consistent with a meta-analysis using a referral and screening population, regardless of detection methods and devices (87%; *κ*: 0.66) [9] and a Japanese referral population (84%) [22]. Another study using the Evalyn brush and Cobas 4800 for ASCUS+ patients showed an agreement rate of 89.2% (*κ*: 0.70) [20], whereas a study using these devices to evaluate patients with premalignant lesions, cervical carcinoma, and carcinoma suspicion reported an agreement rate of 91% (*κ*: 0.64) [19]. In the present study, NILM/HPV-positive cases differed from these studies but showed a similar agreement rate. HPV-16/-18 is associated with malignant tumors [4, 23]. Therefore, it is important that self-sampling HPV-16/-18 results are consistent with those of physician sampling. We found that HPV-16/-18 results showed higher agreement between sampling methods than HPV others, which was consistent with previous reports using the Evalyn brush and Cobas 4800 [20]. These findings demonstrated for the first time that HPV self-sampling using the Evalyn brush and Cobas 4800 agreed with physician sampling for HPV typing in the Japanese population.

Cases showing different results between sampling methods complicate the introduction of HPV self-sampling in screening. Most discrepant HPV tests involved NILM, and our results showed for the first time that discrepant cases using the Evalyn brush and Cobas 4800 in a Japanese population did not involve dysplasia. Inconsistencies in these cases associated with HPV testing between self- and physician sampling might be explained by self-sampling detecting HPV only in the vagina with no infected hrHPV in the cervix [10]. A previous study using Cobas 4800 showed that a high load of non-HPV16/HPV18 associated with the presence of dysplasia in physician-collected hrHPV. In cases without dysplasia, viral load was lower [24]; therefore, A lower load of hrHPV infection in cervix shed cells was impossible for their detection by self-sampling.

The sensitivity of CIN2+ detection by self-sampling was 100% and the same as physician sampling. A previous study evaluating sampling using the brush and PCR-based hrHPV testing by women referred for colposcopy due to abnormal cervical smear and/or post-coital bleeding with normal cytology showed a CIN2+-detection sensitivity of 93% for self-sampling and 91% for physician sampling [25]. Another study evaluating the same methods in women that were previously hrHPV+ showed a CIN2+-detection sensitivity of 100% for both self- and physician sampling [26]. The results of the present study were consistent with these findings. Additionally, a study reported a lower CIN2+-detection sensitivity with the Evalyn brush and HC methods for self-sampling relative to physician sampling in a Japanese referral population [27]. In the present study, the Evalyn brush and PCR-based method for self-sampling showed the same CIN2-detection sensitivity as physician sampling, supporting the superiority of PCR-based HPV testing for self-sampling in a Japanese population.

Previous studies indicated that CIN2+-detection sensitivity by self-sampling exceeded that of cytology in a referral population [15]; however, the present study revealed self-sampling as showing similar CIN2+-detection sensitivity to cytology, possibly due to the small number of CIN2+ cases in our study. Moreover, we found that both self- and physician sampling showed a lower CIN2+-detection specificity than cytology, reflecting either the possible presence of hrHPV infections not yet progressing to CIN2 or detection of vaginal hrHPV infection [10].

Here, samples collected with the Evalyn brush remained dried until transfer to a ThinPrep vial. Our result showed that the number of days from self-sampling to HPV testing did not influence discrepancies in HPV testing. The Evalyn brush is reportedly stable for the measurement of HPV DNA PCR results for up to 32 weeks [28], suggesting that discordant cases are independent of preservation methods.

Previous studies of HPV self-sampling targeted possible applications for cervical cancer screening; however, the high NPV value associated with HPV self-sampling for CIN2+ indicated that self-sampling might be useful for following up potential high-risk groups previously identified as HPV+. A previous study following HPV+ women reported an NPV for HSIL or worse was 98.8% (95% CI 91.6–99.8%) in self-sampling and support self-sampling for exclusion of the disease during follow-up of HPV-positive women [29]. In the present study, the population had a high hrHPV+ rate and included follow-up patients that were previously HPV+. Using pathologic examination as the standard for NPV in our study revealed self-sampling as having a 100% NPV for CIN2+ detection, suggesting that a population at high risk for HPV might not require or delay regular visits upon acquisition of an HPV− result from self-sampling.

This study has some limitations. We examined a small number of referral cases, and sampling was not performed in a screening setting; however, to ensure the feasibility of HPV self-sampling using the Evalyn brush and Cobas 4800, it was necessary to evaluate match rates and CIN2+-detection sensitivity in a Japanese referral population. Therefore, studies in a Japanese screening population are needed. Additionally, HPV self-sampling was performed in a hospital and not at home, which would be the case in a screening setting. HPV-infection status can be affected not only by recent sexual acquisition or re-infection but also from recurrent detection of a controlled or latent infection [30]. Therefore, we attempted to determine whether physician and self-sampling were equally accurate when performed within the same timeframe. Two previous studies evaluating the Evalyn brush and Cobas 4800 involved home-based self-sampling, with their HPV concordance and CIN2+-detection rate consistent with those of our study involving hospital-based self-sampling [18, 20]. Similar results might be obtained from home-based self-sampling in Japanese populations.

In conclusion, HPV self-sampling using an Evalyn brush and Cobas 4800 showed good agreement with physician sampling in a Japanese population. Moreover, the sensitivity of CIN2+ detection by self-sampling was as high as that for physician sampling using Cobas 4800 in a Japanese population. These results suggest that HPV self-sampling using the Evalyn brush and Cobas 4800 PCR-based testing methods might be efficacious for cervical cancer screening in Japan.
